# Monitoring of Astroviruses, Brno-Hantaviruses, Coronaviruses, Influenza Viruses, Bornaviruses, Morbilliviruses, Lyssaviruses and Pestiviruses in Austrian Bats

**DOI:** 10.3390/v16081232

**Published:** 2024-07-31

**Authors:** Sasan Fereidouni, Sinan Julian Keleş, Kore Schlottau, Zoltán Bagó, Guido Reiter, Markus Milchram, Bernd Hoffmann

**Affiliations:** 1 Research Institute of Wildlife Ecology, University of Veterinary Medicine Vienna, 1160 Vienna, Austria; keles.julian@gmail.com; 2 Institute of Diagnostic Virology, Friedrich Loeffler Institut, Federal Research Institute for Animal Health, D-17493 Greifswald-Insel Riems, Germany; kore.schlottau@fli.de (K.S.); bernd.hoffmann@fli.de (B.H.); 3 Austrian Agency for Health and Food Safety Ltd. (AGES), Institute for Veterinary Disease Control, 2340 Mödling, Austria; zoltan.bago@ages.at; 4 Austrian Coordination Centre for Bat Conservation and Research (KFFÖ), 4060 Leonding, Austria; guido.reiter@fledermausschutz.at; 5 Institute of Zoology, BOKU University, 1180 Vienna, Austria; markus.milchram@fledermausschutz.at

**Keywords:** bat monitoring, Austria, influenza viruses, bornaviruses, morbilliviruses, lyssaviruses, pestiviruses, astroviruses, Brno-hantaviruses, coronaviruses

## Abstract

Here, we report the results of a monitoring study of bat viruses in Austria to strengthen the knowledge of circulating viruses in Austrian bat populations. In this study, we analyzed 618 oropharyngeal and rectal swab samples from 309 bats and 155 pooled tissue samples from dead bats. Samples were collected from 18 different bat species from multiple locations in Austria, from November 2015 to April 2018, and examined for astroviruses, bornaviruses, coronaviruses, hantaviruses, morbilliviruses, orthomyxoviruses (influenza A/C/D viruses), pestiviruses and rhabdoviruses (lyssaviruses) using molecular techniques and sequencing. Using RT-qPCR, 36 samples revealed positive or suspicious results for astroviruses, Brno-hantaviruses, and coronaviruses in nine different bat species. Further sequencing revealed correspondent sequences in five samples. In contrast, none of the tested samples was positive for influenza viruses A/C/D, bornaviruses, morbilliviruses, lyssaviruses, or pestiviruses.

## 1. Introduction

Bats are able to establish various ecological niches around most parts of the globe except the polar regions. Their distribution ranges from deserts to forests and from caves to cities, and using caves, bridges, and buildings as preferred roosting sites [1,2]. Bats have a great impact on various ecosystems due to their special adaptations and life traits. Examples of these include their ability to fly, thus being able to migrate distances of more than 2000 km [3,4]; extreme longevity (of more than 30 years) compared to non-flying mammals of similar body size; daily torpor; and seasonal hibernation [5]. As major nocturnal aerial predators, they significantly contribute to natural crop pest control [6,7,8]. They have a great impact on reforestation and agriculture via seed dispersal and pollination activity [9]. Bats comprise more than 1400 known species, hence representing close to 20% of mammalian species diversity [10]. Europe’s bat communities comprise 47 different species [11], of which 31 species have been recorded in Austria [12,13,14,15].

Bats (Order: Chiroptera) are generally considered reservoirs of a wide range of viruses, partly with zoonotic potential for public health [9,16,17,18,19]. Bats may harbor and disperse highly infectious viruses without evidence of a clinical infection [20]. It has been hypothesized that flight triggers a fever-like response, elevating body temperature and metabolic functions, which, compared to viral infections, may cause proportionally higher metabolic costs for bats [21]. This may consequently accelerate the potential of bats’ immune systems to control viral replication more efficiently than other mammals [22]. Therefore, bats are considered to be unique viral hosts because viruses that are adapted to a fever response in bats potentially are still virulent to other hosts [21]. Bats have been associated with various emerging infectious diseases (EIDs), such as severe acute respiratory syndrome (SARS); Middle East respiratory syndrome (MERS); and Ebola, Marburg, Hendra, and Nipah virus [18,23,24,25,26,27,28].

Meanwhile, a coronavirus disease 2019 (COVID-19) pandemic was declared by the World Health Organization (WHO) on 11 March 2020, and, since its starting as an epidemic in China, due to the close genetic relationship between the novel coronavirus and the coronaviruses responsible for the SARS outbreak of 2003, bats and/or pangolins were considered as a potential source of the infection [29].

Among European bats, a variety of pathogens have been detected recently. At least seven different rhabdoviruses of the genus Lyssavirus, including European bat lyssavirus types 1 and 2, were detected in several European countries [30,31,32,33,34] (https://www.who-rabies-bulletin.org, accessed on 30 April 2024). Lyssaviruses are one of the most important viral zoonoses worldwide, and several species of carnivores, apart from bats, act as reservoirs. European bat lyssavirus types 1 and 2 (EBLV-1 and EBLV-2) are widely distributed throughout Europe; EBLV-1 was mainly detected in serotine (Eptesicus serotinus) and Isabelline serotine (Eptesicus isabel-linus) bats, and EBLV-2 mainly in Daubenton’s bats (*Myotis daubentonii*) and Pond bats (*M. dasycneme*) [31,34]. It is important to mention that although lyssaviruses are present in European bats, the number of reported bat-related human cases of rabies is very low [35]. Other viruses found in European bats were flavivirus strains, identified in Germany [36]; herpesviruses among the bats in the Iberian Peninsula [37]; and a novel rotavirus in Serbia [38]. Astroviruses were described in bats in the Czech Republic [39] and Hungary [40]; a filovirus was found in Hungary [41] and in Italy [42]; paramyxoviruses were recorded in Germany; and a variety of coronaviruses were detected in many European countries, such as Germany [43], Luxembourg [44], Italy [45], France [46], Spain [47], Russia, UK, and the Netherlands [48,49,50,51]. A hantavirus strain was identified in the Czech Republic [52].

Coronaviruses are a group of enveloped, positive sense, single-stranded RNA viruses that belong to the family Coronaviridae and are classified into four genera, designated as alpha, beta, gamma, and delta coronaviruses. Gamma and delta coronaviruses are predominately found in avian species, whereas alpha and beta coronaviruses infect mammals, including humans [53]. Hantaviruses are a group of RNA viruses belonging to the family Hantaviridae. These are currently divided into four genera, namely Orthohantavirus, Thottimvirus, Mobatvirus, and Loanvirus, and bat-associated hantaviruses have been detected in Asia, Africa, and Europe. Astroviruses (AstVs) are common gastrointestinal pathogens of animals and humans that may cause gastroenteritis and diarrhea. Astroviruses were mostly found in apparently healthy bats without causing any pathogenic effects [40,54]. Bats may host diverse strains of AstVs from a wide range of species, which indicates a high rate of interspecies transmission and the important role of bats in the ecology and epidemiology of these viruses [55]. Bats have been identified to be able to infect humans via intermediate hosts, such as horses, pigs, civets, and non-human primates [56]. Additionally, an increase in potential encounters via anthropogenic activity in the habitats of bats [18] adds to a higher probability of spillover events. Therefore, understanding the role of bats as reservoirs of zoonotic and emerging viruses is inevitable. Previous studies show a prevalence of different viral infections in European bats. These include viruses of the following families: Adeno-, Astro-, Borna-, Bunya-, Corona-, Filo, Hepe-, Herpes-, Orthomyxo-, Papilloma-, Paramyxo-, Reo-, and Retroviridae [19,57]. Tropical regions with a high wildlife biodiversity and land use change are prone to be EID “hotspots”. However, due to global warming, European countries and cities outside of the tropical regions may be considered high-risk areas for the occurrence of EIDs in the near future [16]. Studies, evaluating the potential risks of viral diseases in Austria are lacking. Therefore, here, we focus on monitoring Austrian bat species for several viral infections such as astro-, borna-, hanta-, corona-, influenza-, morbilli-, pesti-, and lyssaviruses. We aimed to gain more information regarding the distribution of the viral pathogens mentioned above as well as the putative role of bats as carriers and potential vectors. This study also emphasizes the importance of the ecology and epidemiology of those pathogens and provides data on the pre-pandemic spread of bat coronaviruses circulating in European bat populations.

## 2. Materials and Methods

### 2.1. Study Plan

**Figure 1:**
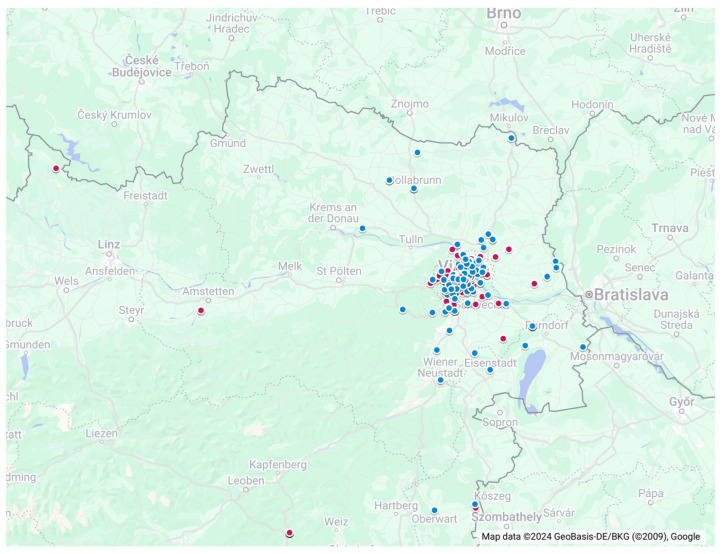
Distribution of collected swab samples (red dots), and organ samples (blue dots) in Austria. A detailed and interactive map of the sample distribution can be accessed via the following link: https://www.google.com/maps/d/u/0/edit?mid=1MBtj9uRBS8C53u-a18jj23MKn2q2IwY (accessed on 10 May 2024).

Samples were collected from 464 bats belonging to 18 species (3 families, 9 genera, Table 1) from November 2015 to April 2018 in six Austrian states: Burgenland, Salzburg, Styria, Upper and Lower Austria, and Vienna (Figure 1). More than 70% of the samples originated from Vienna. The samples were mainly obtained from captured bats after rescue and rehabilitation activities, during monitoring and conservation activities, or from dead-found bats.

Both oropharyngeal and rectal swab samples were collected from each live bat (309 oropharyngeal and 309 rectal swabs). We additionally received pooled samples of various internal organs (heart, lung, liver, spleen, kidney, intestines, and brain) from deceased bats. Rayan swabs (aluminum stick with small viscose tip, diameter 0.9 × 145 mm, article number: 09-816-0001, Nerbe-plus, Germany) were used. The oropharyngeal and rectal swabs were stored separately in cryotubes containing viral transport mediums (PBS + 500 µg enrofloxacin/mL) and maintained at −80 °C after arrival at the laboratory. The suitability of Rayan swabs for sample transmission has been shown previously [58].

The brains of the dead bats were collected separately and were tested for rabies using the fluorescent antibody test (FAT) at the National Reference Laboratory for Rabies of the Austrian Agency for Health and Food Safety (AGES). Bat species were identified based on morphology by experienced bat conservationists. Collaboration with bat conservation societies and rehabilitation centers provided the opportunity for sample collection with minimum impact on Austrian bat populations. No bat was captured exclusively for the current study, and samples were obtained from three different sources, which were the following:

(A) We collected 92 oropharyngeal and rectal swab samples from 46 bats. Bats were captured in the course of monitoring and inventory (population dynamic and biology–ecology) studies with mist nets. Nets were installed in front of caves during autumnal swarming, as well as in foraging areas (i.e., water bodies and woodland). Mist nets with mesh sizes of 20 × 20 mm (monofilament) and 14 × 14 mm (hair net) were used to capture the bats (Ecotone Goc, Sopon, Poland).
(B) Also, 526 oropharyngeal and rectal swab samples were collected from 263 bats that were sent to a bat rehabilitation facility at the University of Veterinary Medicine, Vienna. The bats were captured after rescue activities and subsequently released after rehabilitation.
(C) Lastly, 155 pooled tissue samples were collected between November 2015 and February 2018 during the necropsy of dead-found bats. The brain samples were removed and separately collected for passive surveillance and tested negative for rabies using the FAT method by AGES. The internal organ samples were stored as a pool per individual at −80 °C in plastic containers and were kindly provided for this study.

Swab samples were mainly collected in 2018 from 16 different species, 56% from *Nyctalus noctula*, followed by *Pipistrellus kuhlii* (10%), *Vespertilio murinus* (8.4%), *Pipistrellus nathusii* (5.5%), *Pipistrellus pipistrellus* (4.2%), *Hypsugo savii* (3.2%), *Barbastella barbastellus* (3.2%), and other less representative bat species (Table 1). Organ samples were collected from 13 different species, with most samples (26.5%) from *H. savii*, followed by *N. noctula* (22%), *P. pipistrellus* (11%), *V. murinus* (11%), *P. nathusii* (10.3%), *P. kuhlii* (9%), and other less representative bat species. Approximately 6.5% (10/155) of organ samples were collected in 2015, 31% (48/155) in 2016, 55.5% (86/155) in 2017, and 7.1% (11/155) in 2018. Samples were mainly collected from Vienna and then the Lower Austria states (Figure 1). Bats were handled and cared for in accordance with the Animal Protection guidelines, and legal approval of the sampling had been granted (Ethics Committee Approval ETK-08/02/2018).

### 2.2. Sample Preparation and Nucleic Acid Extraction

Oropharyngeal and fecal swabs were centrifuged at 8000 rpm for 30 s after thawing. The complete medium of approx. 800 µL was then transferred into 1.2 mL Low-Profile 96-Deep-Well Plates (Macherey-Nagel, Düren, Germany). A three samples/well randomly based pooling protocol was then realized by manually transferring a 100 µL medium of each sample into Deep-Well Plates. Then, the primary 96-Deep-Well Plates were frozen at −20 °C.

Internal organ samples and brain from each bat were cut under sterile conditions and pooled into 2 mL microtubes filled with a 5 mm steel bead and 1 mL medium MEM (H) + 1:500 dilution of gentamycin/amphotericin B (Gibco, Life Technologies, Carlsbad, CA, USA). Microtubes were then processed with TissueLyser II for 3 min at 30 Hz (Qiagen, Venlo, the Netherlands). Microtubes were shortly centrifuged, and a 100 µL medium from three samples was pooled into each well of a Deep-Well Plate.

Nucleic acid was extracted from each pool of swab and tissue samples, according to the manufacturer’s instructions. Briefly, 100 µL of sample material was extracted using the NucleoMagVet Kit (Macherey-Nagel, Düren, Germany) on the KingFisher Flex extraction system (Thermo Fisher, Darmstadt, Germany) and eluted in a 100 µL elution buffer. The plates were closed with foil and stored at −20 °C until further processing. Positive detections in pool samples were verified by re-extraction of the individual sample. A positive PCR reactivity was only determined if the individual sample gave a corresponding indication.

### 2.3. Diagnostic Procedure

The viral discovery was performed using qPCR and RT-qPCR with the lab’s internal primers (see below for details), targeting rhabdoviruses/lyssaviruses, orthomyxoviruses/influenza A/C/D viruses, coronaviruses, hantaviruses, astroviruses, morbilliviruses, and bornaviruses. The qPCR- and RT-qPCR-positive samples were considered for short-fragment sequencing.

### 2.4. RT-qPCR

The extracted nucleic acid was analyzed by different RT-qPCRs using the qScript XLT One-Step RT-qPCR ToughMix (Quanta/VWR, Beverly, MA, USA) for influenza virus A, hantaviruses, lyssaviruses, bornaviruses, and influenza viruses C/D; the AgPath One-step RT-PCR Kit (Ambion, Life Technologies, Carlsbad, CA, USA) for morbilliviruses; or the SensiFAST SYBR No-ROX One-Step Kit (Bioline, London, UK, Article No.: BIO-98002) for astroviruses, ß-coronavirus, and pestiviruses (Table 2).

Mostly, 9-well PCR plates (AB-0600-L, Thermo Fisher Scientific) were used to create the master-mix plate. Then, by using the manual pipetting system (Liquidator™ 96, Mettler-Toledo Inc., Columbus, OH, USA) master mixes were transferred into the final master 384-well PCR plates (Hard-Shell^®^ PCR Plates 384-well, Catalog# HSP3801; BIO-RAD Laboratories, Hercules, CA, USA). The same procedure was performed on all sample pool plates.

The master pool plates were centrifuged using the Plate Centrifuge (Eppendorf Centrifuge 5804; Eppendorf, Hamburg, Germany) shortly before analysis (C1000™ Thermal Cycler or CFX384™ Real-Time System, BIO-RAD Laboratories, Hercules, CA, USA). Then, the analyses were performed according to the individual protocol (see below) using a PCR program/software (Bio-Rad CFX Manager 3.0).

In all tests, negative RNA isolation controls (RICs), positive PCR controls (PCs), and negative template controls (NTCs) were added. For testing the inhibition-free transcription and amplification, a heterologous internal control system (IC2-RNA) based on the EGFP gene was used [59]. For testing the successful nucleic acid extraction, different ß-actin qPCR systems were applied [60,61,62].

**Table 2:** qPCR and RT-qPCR protocols used in this study.

Virus Genus/Species	Protocol Name	Oligo Name	Oligo Sequence (5′ → 3′)	Fragment Size (Gene)	AC	References
Influenza A virus	IAV-PB1	IAV-PB1_120F	CAT TTG AAT GGA YGT CAA YCC GA	152 bp (PB1)	A	[63,64]
		IAV-PB1_271R	CTG TTD ACY GTG TCC ATD GTG TA			
		IAV-PB1_247FAM	FAM-CCW GTY CCY GTY CCA TGG CTG TA-BHQ1			
Influenza C/D virus	Pan-IVC/D-Mix1	IVC/D PB1-713F	GCC AAA GAY GGR GAA AGA GG	152 bp (PB1)	A	in-house
		IVC/D PB1-864R	TTC TCA TTD CCV CCA ACA GG			
		IVC/D PB1-779FAM	FAM-CCA TTY TCA AAA ATT GTD GAA ACT KTA GCA CA-BHQ1			
Bornavirus	Pan-Borna-Mix7.2	Borna-1319F	CGC GAC CMT CGA GYC TRG T	211 bp (P)	A	[65]
		Borna-1529R	GAC ARC TGY TCC CTT CCK GT			
		Borna-1471.2FAM	FAM-AAG AAY CCH TCC ATG ATC TCM GAY CMA GA-BHQ1			
Hantavirus	Hanta-Brno-Mix1	Hanta-Brno-234F	GAT TAG TTA GAG GTA ATT GGT TAC A	109 bp (POL)	B	[66]
		Hanta-Brno-342R	GTT AGA GGT AAT TGG TTA CAA GGA			
		Hanta-Brno-274FAM	FAM-TCM GAG AAT AAT TCA CTC CAA ACT-BHQ1			
Morbillivirus	Pan-Morbilli-Mix21	Morbilli-1072F	TCA RTT CAG AAC AAR TTY AGT GC	103 bp (NP)	C	In-house
		Morbilli-1174R	CAA GTT YAA ICC YCC CAT KGA			
		Morbilli-1111FAM	FAM-CTM TGG AGY TAT GCB ATG GGW GT-BHQ1			
Lyssavirus	Lyssa-N-MGB-FAM-Mix1v3+LBV	Lyssa_N_1-F-a	ACG CTT AAC AAC AAA ATC ATA AAA	165 bp (NP)	D	In-house
		Lyssa_N_1-F-b	ACG CTT AAC AAC AAA ACC AAA GAA			
		Lyssa_N_1-F-c	ACG CTT AAC AAC AAG ATC AAA GAA			
		Lyssa_N_1-F-d	ACG CTT AAC AAC AAA ATC ATA GAA			
		Lyssa_N_1-F-e	ACG CTT AAC AAC AAA ATC AGA GAA			
		Lyssa_N_1-F-f	ACG CTT AAC AAC CAG ATC AAA GAA			
		Lyssa_N_1-F-g	ACG CTT AAC AAC AAA ATC AAA GAA			
		Lyssa_N_1-F-h	ACG CTT AAC GAC AAA ATC AGA GAA			
		Lyssa_N_1-F-i	ACG CTT AAC AAC AAA AAC AAA AAA			
		Lyssa_N_1-F-j	ACG CTT AAC GAC AAA ACC AGA AAA			
		Lyssa_N165-146R	GCA GGG TAY TTR TAC TCA TA			
		Lyssa_N_143-R-a	GCA GGA TAT TTG TAC TCA TA			
		Lyssa_N_143-R-b	GCA GGG TAC TTG TAC TCA TA			
		Lyssa_N_143-R-c	GCA GGG TAC TTG TAC TCA TG			
		Lyssa_N_143-R-d	GCA GGG TAT TTG TAC TCA TA			
		Lyssa_N_143-R-e	GCG GGG TAT TTG TAC TCA TA			
		Lyssa_N_143-R-f	AGC CGG GTA TTT GTA CTC GTA			
		Lyssa_N_85_LBV_FAM-Taq	FAM-GAT TGT KTT YAA RGT TCR TAA TCA G-BHQ1			
		Lyssa_N_53-FAM-MGB	FAM-AAA TGT AAC ACC YCT ACA ATG GA-MGBNFQ			
Pestivirus	Pan-Pesti-NS5B-Assay 2	Pesti-11453-F	ACA GCM ATR CCA AAR AAT GAG AA	154 bp (POL)	E	[67]
		Pesti-11607-R	TTT CTG CTT TAC CCA VTT RTA CAT			
Coronavirus	Pan Corona-beta Mix 10 (SYBR Green)	CoV-beta-17106F	TCC WCA MCT TAT GGG TTG GGA	454 bp 447 bp 238 bp 231 bp (POL)	F	In house
		CoV-beta-17113F	CCT TAT GGG TTG GGA YTA YCC			
		CoV-beta-17344R	TAG CAT AAG CAG TDG TDG CAT C			
		CoV-beta-17560R	GCC ATC ATC ASA MAR AAT CAT CAT			
Astrovirus	Pan-Astro-K22 (SYBR Green)	Astro-K22-3162-F	CAC GTT TTG ATG GBA CVA THC C	411 bp (ORF1b) 170 bp (ORF2)	G	In house
		Astro-K22-3573-R	TCA GGY TTR ACC CAC ATD CCR AA			
	Pan-Astro-K24 (SYBR Green)	Astro-K24-3714-F	AAT TWG CCC TCT RTG GGA ARC T			
		Astro-K24-3884-R	TCT TTG GTC CKC CCC TCC A			

Amplification conditions (AC): A: 10 min 50 °C, 1 min 95 °C, 45 cycles of 10 s 95 °C, 30 s 57 °C, and 30 s 68 °C. B: 10 min 50 °C, 1 min 95 °C, 45 cycles of 10 s 95 °C, 30 s 58 °C, and 30 s 68 °C. C: 10 min 45 °C, 10 min 95 °C, 45 cycles of 15 s 95 °C, 20 s 56 °C, and 30 s 72 °C. D: 10 min 50 °C, 1 min 95 °C, 45 cycles of 10 s 95 °C, 20 s 57 °C, and 20 s 68 °C. E: 10 min 45 °C, 2 min 95 °C, 40 cycles of 5 s 95 °C, 15 s 56 °C, 15 s 72 °C, and 15 s 78 °C; then, 1 min 95 °C, 1 min 55 °C, and melting curve for 10 s from 55–95 °C. F: 10 min 45 °C, 2 min 95 °C, 45 cycles of 5 s 95 °C, 15 s 58 °C, 15 s 72 °C, and 15 s 78 °C; then, 1 min 95 °C, 1 min 55 °C, and melting curve for 10 s from 55–95 °C. G: 10 min 45 °C, 2 min 95 °C, 45 cycles of 10 s 95 °C, 10 s 60 °C, and 30 s 72 °C; then, 1 min 95 °C and 1 min 55 °C, and melting curve for 5 s at 55–95 °C.

### 2.5. Sequencing

Short-fragment sequencing was performed for the RT-qPCR-positive samples using the PCR product. The fragment size of the positive samples was analyzed in the agarose gel, and appropriate amplicons were extracted, purified, and sequenced using the respective PCR primers with the standard procedure of Sanger sequencing. Briefly, PCR products were analyzed using 1.5% agarose gel with ethidium bromide. Band size was calculated using a 100 bp ladder (Thermo Fisher Scientific, Darmstadt, Germany). After running at 100 V for 50 min, the agarose gel was analyzed under UV light, and bands were cut out. PCR products were extracted using the QIAquick Gel Extraction Kit from Qiagen (Hilden, Germany) according to the manufacturer’s instructions. PCR products with the respective primer were submitted to Eurofins Genomics (Ebersberg, Germany) for sequencing. The generated sequences were analyzed using Geneious software v10.2.3 (Biomatters Ltd., Auckland, New Zealand).

### 2.6. Fluorescent Antibody Test (FAT)

The brains of the dead bats were collected separately and tested for rabies by FAT according to WOAH [68] and using FITC Anti-Rabies Monoclonal Globulin as the primary antibody (Fujirebio Diagnostics Inc., Seguin, TX, USA).

## 3. Results

A pooling policy revealed very robust results, and individual samples out of the positive pools were tested by additional qPCR and RT-qPCR and sequencing to reveal the individual positive samples. In total, 36 swab and organ samples revealed positive or suspicious RT-qPCR results, and we were able to sequence a part of the genome (241–431 bp) of 5 out of the 36 samples to find the genetic relationship of the Brno-hantaviruses, coronaviruses, and astroviruses compared to common European genetic diversity of those viruses (Table 3).

Alpha and beta coronaviruses and astroviruses were found in both swab samples and tissue samples, while Brno-hantaviruses were found only in one tissue sample (BH 35/18_760) collected from a dead *N. noctule* in Bruck an der Leitha (Lower Austria) in 2017, and that sample also revealed positive short-fragment sequencing results (241 and 431 bp with GenBank numbers PP976056 and PP976057), with 86% identity to Brno loanvirus isolate BH08/16-23 glycoprotein (GenBank number OM912838) and 97.3% identity to Brno virus isolate 11/2013/CZE RNA-dependent RNA polymerase (RdRp) gene (GenBank number KR920360), respectively (Table S1). The RT-qPCR analysis of the samples indicated sufficient viral load (with a Ct value equal to 27.6).

Twenty-one organ samples from different species (Table 3), and three fecal swab samples from two *P. kuhlii* and one *P. nathusii* tested positive for coronaviruses (with Ct values 30.9, 34.2, and 36.5); however, only three swab samples generated short-fragment (409 bp) sequences (GenBank numbers PP976053-PP976055), with more than 98% identity to alpha and beta coronaviruses from Italy (GenBank number MH938450) and the Netherlands (GenBank number OQ348393), respectively (Table S1).

Finally, one oral swab sample from *N. noctula* and ten organ samples from different species (Table 3) were positive for astroviruses, and the swab sample revealed short-fragment (387 bp) astrovirus sequence (GenBank number PP976052), with more than 99% identity to astrovirus isolate Bat_AstV-54_2-2018_POL RdRp gene (GenBank number MW399239).

Although all three beta coronavirus RT-qPCR and sequencing-positive samples originated from fecal samples, the only astrovirus-positive sample from which the sequence of the virus was successfully obtained originated from an oral swab sample.

No swab and/or tissue samples were positive for influenza A, C, and D viruses; morbilliviruses; lyssaviruses; or pestiviruses; however, one fecal swab sample collected from a common noctule was positive RT-qPCR for bornaviruses with a Ct value of 34.6; however, as we could not sequence the virus further, we considered it as questionable.

The highest number of positive samples per species originated from *P. nathusii*, *N. noctula*, *P. kuhlii*, and *H. savii*.

## 4. Discussion

The important role of bats as a reservoir for different viruses, including potentially zoonotic and emerging viruses, is well known from intensive investigations from many regions of the world. The surveillance of viral bat infections, especially viruses that may be of human health concern, is becoming increasingly important. This knowledge will facilitate risk assessments concerning many bat-related conservation and research activities as well as the epidemiologic tracing of epidemics and pandemics, such as finding the source of the COVID-19 pathogen. However, little information is available about the circulation of different important viruses in Austria, and this is the first comprehensive study in this field.

Interestingly, we could not identify any lyssavirus positive among swab and organ samples (although the organ samples were screened twice, once previously by AGES), using different molecular methods and a wide range of RT-qPCR assays. This could be caused by the bat species bias sampling during this study. The serotine bat is assumed as the main reservoir for EBLV-1, and only seven serotine bats were sampled during this study. Recently, the first EBLV-1 case was detected in Lower Austria; therefore, further monitoring studies on a larger number of bats are necessary to obtain more information about the presence of lyssaviruses in Austria (https://www.who-rabies-bulletin.org, accessed on 30 April 2024; https://www.ages.at/en/human/disease/pathogens-from-a-to-z/rabies, accessed on 30 April 2024).

We tested the samples for coronaviruses, and we found RT-qPCR-reactive samples in different species, including *P. nathusii*, *P. kuhlii*, *N. noctula*, *H. savii*, *Plecotus austriacus*, *B. barbastellus*, *V. murinus*, and *M. emarginatus*. However, we were able to obtain short-fragment sequences of alpha and beta coronaviruses only in fecal swab samples from two *P. kuhlii* and one *P. nathusii*, respectively, and no oral swab revealed positive results. Interestingly, *P. nathusii* revealed the highest numbers of swab- and tissue-positive results in RT-qPCR (Table 3).

Bat-associated hantaviruses have been detected previously in Europe, and recently, a novel hantavirus (Brno loanvirus, BRNV) was identified in *N. noctula* in the Czech Republic; therefore, we screened bat samples for hantaviruses, and we could identify a Brno-hantavirus-positive sample collected from *N. noctula* among 207 samples collected from the same species [66].

We detected the positive molecular results for AstVs in samples from several species, including *N. noctula*, *P. kuhlii*, *P. nathusii*, *H. savii*, *V. murinus*, *E. serotinus*, and *P. pipistrellus*, which could only be confirmed via sequencing of the virus in an oral swab sample collected from *N. noctula*. AstVs and many other RNA viruses are shed actively via saliva, and this may facilitate the bat-to-bat transmission of the virus, especially as all bat species in this study are highly social, form dense colonies, and show grooming behavior. Bat workers and those who feed the bats in rehabilitation centers should be aware of this phenomenon and avoid facilitating the bat-to-bat transmission of the virus.

Bornaviruses cause fatal encephalitis in several mammalian species, including sheep, horses, and humans. Although a study in Germany has indicated no evidence of infection of bats with Borna disease virus 1 [69], we analyzed the samples to detect any potential positive ones. We only found one weakly reactive fecal swab sample, and due to a lack of sequence data, this result cannot be confirmed; we may investigate more samples using probe-based RT-qPCR in the future.

The discovery of the genome of two new influenza A-like H17 and H18 [70,71] influenza A viruses (IAVs) in yellow-shouldered bats (*Sturnira lilium*) in Guatemala and a distinct H9 lineage of H9 subtype viruses in Egypt [72] drew attention to bats as putative host species or even reservoirs of unknown influenza viruses. Therefore, influenza A/C/and D viruses also were investigated in our study; however, no positive sample was identified. No positive influenza A virus was identified in samples previously collected from Germany, Romania, and the Czech Republic between 2002 and 2013 [73], and this confirms our previous theory that European bat species may not act as a susceptible host for influenza viruses.

*N. noctula* is overrepresented in this study, with 44.6% (207/464) of samples collected from this species, harboring astroviruses, coronaviruses, and Brno-hantaviruses. The next common species was *H. savii*, with approximately 11% of samples originating from this species, followed by *P. kuhlii* representing 9.7% of samples (45/464), which included two out of the three coronaviruses sequencing-positive samples; and *V. murinus* accounted for 9.3% (43/464). The fifth highest number of samples per species originated from *P. nathusii*, with 7.1% (33/464); here, one swab sample revealed a partial sequence of coronaviruses, although eight tissue samples had positive RT-qPCR results.

In summary, the first screening of bat samples from Austria for different virus groups was successfully performed. In principle, both swab samples and tissue samples were suitable for investigation. The detection of astroviruses, hantaviruses, and coronaviruses could be partially confirmed by partial sequencing, but not all positive samples revealed a corresponding sequence. The low viral load of the positive samples could be the reason for the low success of the Sanger sequencing. SYBR Green qPCR assays can have a high diagnostic sensitivity due to the lack of a detection probe. This high sensitivity harbors the risk of non-specific reactions. An absolutely reliable diagnostic statement, especially with SYBR Green qPCR assays, can only be confirmed by partial sequencing. Nevertheless, the positive partial sequences of the corona-, astro-, and hantaviruses confirm the general functionality of the screening procedure. The success and experience gained from these studies should form the basis for further, possibly more comprehensive screening studies. Based on the available data, a conclusive evaluation of only PCR-positive samples without a confirmed sequence is very difficult or impossible. A conclusive positive result can only be defined for samples with a confirmed and unambiguous sequence. Further analyses, including confirmation tests with various alternative PCR systems, were not possible within the scope of the project. The main limitation of this study may be using the SYBR Green qPCR assays because of the need for future sequencing for conformation, and using probe-based qPCR assays will increase the reliability of the tests.

## Figures and Tables

**Table 1:** Total number of sampled bat species in this study (each swab sampling included separate oral and rectal swabs).

Bat Species	English Name	Swab Sampling	Tissue Sampling	No. of Animals
*Rhinolophus ferrumequinum*	Greater horseshoe	3	0	3
*Rhinolophus hipposideros*	Lesser horseshoe	2	0	2
*Myotis daubentonii*	Daubenton’s bat	2	0	2
*Myotis mystacinus*	Whiskered bat	2	4	6
*Myotis emarginatus*	Geoffroy’s bat	0	2	2
*Myotis myotis*	Greater mouse-eared	0	1	1
*Nyctalus noctula*	Common noctule	173	34	207
*Nyctalus leisleri*	Lesser noctule	1	0	1
*Eptesicus serotinus*	Serotine bat	5	2	7
*Vespertilio murinus*	Parti-coloured bat	26	17	43
*Pipistrellus pipistrellus*	Common pipistrelle	13	17	30
*Pipistrellus pygmaeus*	Soprano pipistrelle	3	0	3
*Pipistrellus nathusii*	Nathusius’s pipistrelle	17	16	33
*Pipistrellus kuhlii*	Kuhl’s pipistrelle	31	14	45
*Hypsugo savii*	Savi’s pipistrelle	10	41	51
*Plecotus austriacus*	Grey long-eared bat	6	5	11
*Barbastella barbastellus*	Western barbastelle	10	1	11
*Miniopterus schreibersii*	Common bent-wing bat	5	0	5
Unknown species		0	1	1
		309	155	464

**Table 3:** Results of RT-qPCR (and Ct values) and sequencing-positive samples collected from different bat species.

Viruses	Bat Species	Swab Samples			Organ Samples		
		RT-qPCR Positive	Ct Values	Confirmed by Sequencing	RT-qPCR Positive	Ct Values	Confirmed by Sequencing
Hantaviruses	*N. noctula*	-	-	-	1	27.6	1
Coronaviruses	*P. kuhlii*	2	30.9, 34.2	2	2	33.4, 33.9	-
	*P. nathusii*	1	36.5	1	8	31.2, 31.4, 31.9, 33.3, 33.6, 33.7, 34.3, 34.6	-
	*H. savii*	-	-	-	5	31.5, 32.1, 32.6, 34.1, 35.2	-
	*V. murinus*	-	-	-	2	33.4, 35.7	-
	*N. noctula*	-	-	-	1	36.9	-
	*P. austriacus*	-	-	-	1	36.5	-
	*B. barbastellus*	-	-	-	1	35.1	-
	*M. emarginatus*	-	-	-	1	33.1	-
Astroviruses	*N. noctula*	1	37.2	1	2	35.2, 32.4	-
	*P. kuhlii*	-	-	-	3	32.3, 31.9, 35.7	-
	*P. nathusii*	-	-	-	1	31.3	-
	*H. savii*	-	-	-	1	32	-
	*V. murinus*	-	-	-	1	33	-
	*P. pipistrellus*	-	-	-	1	28.6	-
	*E. serotinus*	-	-	-	1	32.6	-

## Data Availability

The geographical area of sampling has been shown in Google Maps and is publicly available.

## Supplementary Materials

The following supporting information can be downloaded at: https://www.mdpi.com/article/10.3390/v16081232/s1, Table S1. Results of sequencing data generated from the positively sequenced samples.
