# Supplementary material for: Monitoring of Astroviruses, Brno-Hantaviruses, Coronaviruses, Influenza Viruses, Bornaviruses, Morbilliviruses, Lyssaviruses and Pestiviruses in Austrian Bats
Source: Viruses. 2024 Jul 31;16(8):1232. doi: 10.3390/v16081232 (PMC11359250; doi:10.3390/v16081232)
Supplement: Supplementary file 1 [file viruses-16-01232-s001.zip › viruses-3034809-supplementary.pdf]

viruses

Table S1. Results of sequencing data generated from the positively sequenced samples.

Viruses	Bat species	Gene	Sequence length	Accession number
Astroviruses	*Nyctalus noctula* (oral swab)	RdRp	387 bp	PP976052
Coronaviruses	*Pipistrellus kuhlii* (fecal swab)	RdRp	408 bp	PP976053
	Pipistrellus nathusii (fecal swab)	RdRp	408 bp	PP976054
	Pipistrellus kuhlii (fecal swab)	RdRp	408 bp	PP976055
Hantaviruses	*Nyctalus noctula* (tissue sample)	Glycoprotein	241 bp	PP976056
		RdRp	429 bp	PP976057
